# PROTACs and Glues: Striking Perspectives for Engineering Cancer Therapy À La Carte

**DOI:** 10.3390/ph18091397

**Published:** 2025-09-17

**Authors:** Jean-Marc Ferrero, Jocelyn Gal, Baharia Mograbi, Gérard Milano

**Affiliations:** 1 Department of Medical Oncology, Antoine Lacassagne Center, University Côte d’Azur, 33 Avenue de Valombrose, 06189 Nice, France; jean-marc.ferrero@nice.unicancer.fr; 2 Department of Epidemiology and Biostatistics, Antoine Lacassagne Center, University Côte d’Azur, 33 Avenue de Valombrose, 06189 Nice, France; jocelyn.gal@nice.unicancer.fr; 3 FHU OncoAge, IHU RespirERA, IRCAN, Inserm, CNRS 7284, U1081, University Côte d’Azur, 06189 Nice, France; baharia.mograbi@univ-cotedazur.fr; 4 Scientific Valorization, Antoine Lacassagne Center, University Côte d’Azur, 33 Avenue de Valombrose, 06189 Nice, France

**Keywords:** PROTACs, molecular glue, artificial intelligence, anticancer drug, personalized therapy

## Abstract

PROTACs are bifunctional small molecules that simultaneously bind a target protein and a component of the ubiquitin–proteasome system, thereby inducing selective degradation of the target. They represent a class of compounds capable of achieving the complete elimination of disease-relevant proteins. Molecular glues, by contrast, enhance existing surface complementarity between an E3 ligase and a target protein, promoting its ubiquitination and subsequent degradation. Both approaches are at the forefront of current efforts to overcome the long-standing challenge of undruggable tumor targets. In this context, AI-based strategies offer a powerful means to accelerate the discovery, optimization, and production of highly selective protein binders, streamlining access to potent degraders and maximizing therapeutic potential. These capabilities open new horizons for targeting a wide spectrum of previously inaccessible molecular pathways involved in cancer progression. Altogether, these advances position PROTACs and molecular glues as transformative agents for personalized oncology, particularly within the emerging paradigm of molecular tumor boards, where tailored therapeutic decisions and tumor-adapted drugs could be made rapidly accessible for a given patient.

## 1. General Principles

### 1.1. Definition of Degraders and Glues

The term PROTACs (proteolysis-targeting chimeras) was first coined by Sakamoto and coworkers in 2001 [1]. The idea behind the original development of PROTAC degraders is based on the well-established knowledge that protein levels in the cell are regulated through the action of the ubiquitin–proteasome system [2]. This system degrades targeted proteins through substrate-specific ubiquitination and recognition. Ubiquitination consists of a three-step process that involves the juxtaposition of three distinct enzymes: ubiquitin-activating enzymes (E_1_), followed by ubiquitin-conjugating enzymes (E_2_), and, finally, substrate-specific ligases (E_3_). Such an ubiquitination mechanism can be recycled to produce a poly-ubiquitin chain attached to the target protein, orienting the marked protein to the 26S proteasome [2]. This intracellular protein destruction process has been hijacked by PROTACs, which simultaneously recruit the E_3_ ligase and the protein of interest, thus creating a favorable proximity between this protein of interest and the E_3_ ligase. PROTACs are thus bifunctional small molecules that can bind both a given targeted protein to be degraded and a component of the ubiquitin–proteasome system. A single PROTAC molecule can degrade multiple copies of its target protein, thus making relatively low concentrations of PROTACs necessary to achieve an expected biological effect [3]. Also of interest is the notion that, while classical small molecules (such as tyrosine kinase inhibitors) are typically used exclusively as inhibitors or activators, PROTACs constitute a class of agents capable of inducing the total elimination of the target [3].

The integration of PROTACs as molecules that promote targeted protein degradation constitutes a promising advance in the fight against cancer. Interestingly, the molecular design of PROTACs enables the integration of binders for ligases and targets; however, this must be viewed against the background of certain challenges associated with the chemical properties of these binders [4]. Continuous combined efforts in academia and industry have enabled the development of hundreds of PROTACs for targets, including kinases and hormone receptors [5].

Basically, the discovery of molecular glues is linked to the elucidation of the mechanism of action of thalidomide. Several decades after withdrawal of this drug from the market due to teratogenicity, thalidomide was reported in 2010 to bind to the substrate receptor protein of a specific E_3_ ubiquitin ligase [6]. Molecular glues are able to enhance pre-existing surface complementarities between an E_3_ ligase and a target protein to induce its degradation following ubiquitination. This mechanistic insight into thalidomide activity corroborates the discovery that this drug and its congeners are capable of developing antitumoral activity in multiple myeloma as molecular glues, inducing the degradation of transcription factors IKZF1 and IKZF3 [7]. Lenalidomide, a derivative of thalidomide, now forms the backbone of treatments for multiple myeloma and myelodysplastic syndrome [8]. Structural biology studies have revealed that the site of fixation for thalidomide and its derivatives on the transcription factors initially identified as their primary targets is also present in other proteins, thus expanding the area of potential targets for this category of molecular glues [9].

### 1.2. PROTACs Versus Glues

With PROTACs in one hand and molecular glues in the other, it is interesting to compare the respective advantages and limitations of these two relatively similar approaches as therapeutic tools for degrading targeted proteins [10]. Although PROTACs offer unique therapeutic opportunities compared with other targeted cancer treatments, they also have some disadvantages which constitute significant issues and challenges [11]. Firstly, there is a major pharmacokinetic concern affecting PROTACs which is linked to their relatively high molecular weight and hence low values for their permeability and solubility, resulting in an unsatisfactory oral bioavailability [12]. There is thus a need to improve the physicochemical properties of PROTACs. The pharmacokinetic characteristics of PROTACs are also complicated by the existence of a “Hook effect”, meaning that at higher PROTACs concentrations, reduced efficacy can be observed [13]. This phenomenon can be explained by the formation at high PROTACs concentration of a binary complex (proteins-PROTAC or E_3_–PROTAC) instead of the expected ternary complex. As a result, this phenomenon limits the pharmacological significance of the maximal concentration of an administrated PROTACs. Additionally, PROTACs can lead to acquired resistance to treatment due to changes in the genome of the main component of the E_3_ ligase complex [14]. In contrast, molecular glues, due to their lower molecular weight, are expected to present superior pharmacokinetic properties compared to those of PROTACs [10]. Specifically, their reduced molecular size facilitates enhanced cellular internalization and improved transportation across the blood–brain barrier, a critical parameter in the management of CNS metastatic disease.

Another point of comparison lies in the respective constraints of development, with a relatively high complexity in PROTACs design, due to the simultaneous binding of the drug to the targeted protein and the ligase. In this respect, out-target effects of PROTACs have been described and are still not fully understood [11,15]. In contrast, glues are chemically simpler than PROTACs as they consist of a single entity with fewer structural constraints. However, glues have less modularity than PROTACs due to their dependency on both a specific target and a corresponding ligase. Consequently, glues have less flexibility than PROTACs in terms of expanding their spectrum of action. Also, PROTACS can degrade several copies of the same target, conferring a prolonged action at low dose. In contrast glues are less able to perform recycling compared with PROTACS [4]. Table 1 summarizes the respective advantages and disadvantages of PROTACs and glues.

## 2. Degraders and Glues in the Context of Personalized Cancer Therapy

### 2.1. Current Status of Personalized Therapy

After briefly covering the main aspects of PROTACs and glues, the next step is to consider this relatively new family of anticancer agents in the context of personalized therapy. Continuous innovation in genomics and the increasing understanding of the cancer genome have enabled precision oncology to be applied to a broad spectrum of cancer types [16]. Unfortunately, extensive prospective studies evaluating the benefit or not of administering an individualized molecular-based therapy (mainly molecular antibodies and kinase inhibitors) as compared to a standard therapy, do not conclude in favor of a personalized treatment impacting positively patient survival [17,18]. One core explanation for these relatively negative conclusions may lie in the particularly low percentage of patients carrying a given pathogenic mutation who could potentially benefit from personalized therapy [19]. Thus, it is estimated that only 15% of cancer drug targets (enzymes and receptors) can be considered as druggable. However, it is difficult to accept that a significant proportion of 85% of targets remain undruggable with, in particular, RAS proteins [20], transcription factors as p53 [21], proteins involved in epigenetic regulation [22]. Moreover, when new targeting drugs reach the market, they are generally accessible to a particularly low proportion of patients [23]. Consequently, further research is necessary to overcome the obstacle of undruggable tumor targets.

### 2.2. Perspectives Offered by PROTACs and Glues

The domain of PROTACs and glues is potentially well-suited to addressing this unmet need, and drugs representative of this therapeutic class are nearing market release [24,25]. However, an expanding spectrum is urgently needed for proteins of interest to be degraded by such drugs [26]. Important advances could come from the transformative role of generative artificial intelligence (AI) leading to the elucidation of novel protein architectures with certain limits concerning protein folding, dynamics and function [27]. On the other hand, there is the expanding universe of enhanced understanding of the biological foundations of tumor progression including metastases, highlighting important key molecular actors of aggressiveness [28]. Interestingly, AI is able to facilitate and accelerate the development of degraders (PROTACs and glues) at different levels [22,29]. AI-based models such as AlphaFold can predict protein structures and multi-metric assemblies with high accuracy, enabling a deeper understanding of protein conformations and potential interaction sites [30]. AI can also be integrated at various stages of complex technological pipelines to specifically rationalize and accelerate the identification and the production of active degraders, thus enhancing screening throughput and target prioritization [29,31]. A key example of this potential is provided by Petzold et al., who computationally identified over 1400 Cereblon immunomodulatory drugs (CRBN–IMiD) compatible neosubstrates, thereby considerably reshaping existing paradigms of substrate recognition [32]. Building on these foundations, Lu et al. demonstrated the ability of AI-based models to generate de novo small-molecule binders with nanomolar affinities and high specificity [33]. Marchand and coworkers brought significant advances in the field by developing deep learning algorithms capable of identifying ligand-binding neosurfaces, enabling the rational design of highly selective protein binders [34]. Gainza et al. introduced a surface fingerprint-based framework for the de novo engineering of protein–protein interactions without relying on co-evolutionary information, broadening the applicability of AI to poorly annotated targets [35]. Interestingly, Ren and coworkers reported an original end to end drug development (a novel small molecule inhibitor) covering target selection, molecule and preclinical testing; all the steps were carried out in a relatively short period of time thanks to AI [36]. The authors successfully applied AlphaFold, combined with a biocomputational platform—PandaOmics [37] and a generative chemistry42 platform [38]. It is worth noting that PandaOmics is an AI-driven platform that integrates multi-omics, biomedical text mining, and clinical data to prioritize therapeutic targets and biomarkers. Importantly, such platforms can accelerate degrader-based therapies, including PROTACs and molecular glues, by identifying undruggable proteins and mapping E3 ligase-substrate interactions. There are other emerging perspectives like the use of DrugFlow, which is an AI-driven platform offering a cloud-based interface to streamline early drug discovery workflow, integrating innovative AI algorithms [39].

**Figure 1:**
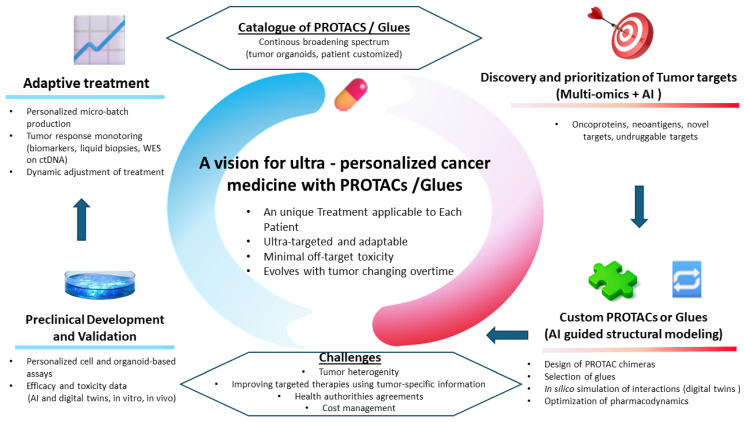
A proposal for a descriptive approach in the dynamics of molecular tumor board management by applying an ultra-personalized cancer medicine based on PROTACs and glues and guided by AI.

The World Wide Innovative Network (WIN) consortium in personalized cancer medicine provides an ongoing vision for innovation, collaboration and global impact in precision oncology [40]. PROTACs and glues can potentially trigger a wave of progress in this context, and their ultimate use as a solution for an optimal personalized therapy could truly revolutionize the practice of precision cancer medicine (Figure 1). The envisaged goal would no longer be to dispose of a panel of conventionally developed targeted drugs, such as monoclonal antibodies or kinase inhibitors, to be classically delivered to patients screened in molecular tumor boards and carrying a specific tumor abnormality [41,42]. Instead and more precisely, the new disruptive strategy herein considered would be to start from the patient specific tumoral characteristics to identify one or several key factors governing tumor progression, and then to shift towards the platform dedicated to the production of truly personalized therapy through the application à la carte of PROTACs and glues (Figure 1). In this context, AI-based approaches like the recent one reported by Ren and co-authors described above [36] should thus accelerate the discovery, identification and production of highly selective protein binders, paving the way for optimal access to degraders with anticipated benefits. The challenge at this level will be the time necessary to deliver the right drug to the right patient. Systematic review and meta-analyses indicate that delaying adjuvant treatment can significantly impact the survival outcome of patients [43]. Realistic figures show, for instance, that 6 to 8 weeks is an optimal delay before applying chemotherapy in cancer [44]. It is worth noting that the new AI-based targeted drug development strategy reported by Ren and coworkers indicates a duration of 30 days for the end to end production of the original drug [36]. In addition, to achieve the objectives of personalization and speed, the application of tumor organoids would be particularly well suited as a preliminary step to validate the concept of such a personalized cancer therapy à la carte [45]. This pre-clinical approach would thus make it possible to create an extensive catalog of potentially clinically applicable PROTACs and glues. It must be underlined that patient-adapted therapy is not new in cancer management. For instance, considerable technologies advances now permit personalized antigen selection and tumor—specific vaccine delivery in a very short time interval [46,47,48]. Likewise, the delivery of CAR-T cell therapy consisting in genetically engineering patient immune cells, represents an access to high technology platforms able to deliver a real-time personalized therapy [49]. More recently, in vivo CAR-T cell generation appeared as a promising alternative to the ex vivo manipulation of these immune cells [50]. This new approach can potentially enlarge the field of application of CAR-T cell therapy. As it is clear that the involvement of drug companies is central for the delivery of personalized vaccines and CAR-T cell therapy, such a boosting role would also be central for the conception, delivery and achievement of personalized degrader therapy. However, critical issues must be taken into account and they cover important aspects like specificity, safety and bioavailability [26]. In this respect, it is not surprising to observe the strong implication of Novartis, a pioneer in CAR-T cell therapy, to co-opt Arvinas, a major actor in degrader discovery, for the development of major clinical trials in the domain of degraders and glues [51].

## 3. Conclusions and Perspectives

Arvinas and Pfizer were able to successfully conduct and complete the clinical development of Vepdegestrant through the trial VERITAC-2 [52]. Vepdegestrant is a PROTAC that targets the estrogen receptor, and the VERITAC-2 trial has reported a significant benefit in event-free survival for ESR1 mutation breast cancer patients who fail to respond to a CDK4/6 inhibitor. Vepdegestrant has been granted Fast Track designation by the FDA. The future prospects of such innovative, personalized medicine should be incorporated into healthcare systems, in collaboration with regulatory agencies and through optimized strategies [53], once the proof of concept becomes available. As a promising paradigm for drug discovery, PROTACs and glues attract growing attention from both academia and pharmaceutical companies. There are, however, critical issues to be addressed for the optimal clinical use of this class of drugs, including the high molecular weight of PROTACs and resulting pharmacokinetic complications, such as poor oral bioavailability. Challenging resistance mechanisms have also been reported for PROTACs and glues. They mainly concern point mutations for the PROTAC target and missense mutations for the engaged E_3_ ligase [54]. These limitations may constitute hurdles in the clinical use of PROTACs and glue, counterbalancing their intrinsic advantage in degrading what are still considered to be undruggable targets. The development of PROTACs and glues is also time-consuming, due to the inherent complexity in designing efficient molecules. But we are entering a new era of rapid technological evolution in both structural and computational biology, primarily driven by the growing, spectacular implications of generative AI (Figure 1). This context creates the conditions for spectacular improvements in accelerated PROTAC and glue development, encompassing all steps from target identification and draggability with E_3_ ligases to assay development and hit history [55]. Last but not least, there remains a certain number of obstacles to be overcome in order to realistically incorporate these motivating perspectives for cancer therapy “à la carte” based on the use of an end to end real time production of PROTACs and glues for individual patients in molecular boards care. These difficulties mainly concern logistical and regulatory aspects, in addition to the intrinsic costs [56]. It is clear that incorporating AI technologies in current regulatory health agencies’ practices raises notable difficulties to be overcome and that condition large scale testing and a more generalized use of an end to end engineering of cancer therapy à la carte [57].

## Figures and Tables

**Table 1:** Respective characteristics and properties of PROTACs and glues.

Criteria	PROTACs	Glues
Conception	Complex	Less predictable
Mechanism of action	Bifunctional	Monofunctional
Size	High (>800 Da)	Low (Inf 500 Da)
Modularity	High	Low
Targeting capacity	Large	Restricted
Conception	Complex	Less predictable
Pharmacokinetics properties	Unfavorable	Favorable
Oral absorption	Erratic	Satisfactory
CNS penetration	Low	High
Prolonged action	High capacity	Low capacity

## Data Availability

Not applicable. There is no data or materials involved in this review.
